# Initial productive and latent HIV infections originate in vivo by infection of resting T cells 

**DOI:** 10.1172/JCI171501

**Published:** 2023-11-15

**Authors:** Stephen W. Wietgrefe, Jodi Anderson, Lijie Duan, Peter J. Southern, Paul Zuck, Guoxin Wu, Bonnie J. Howell, Cavan Reilly, Eugène Kroon, Suthat Chottanapund, Supranee Buranapraditkun, Carlo Sacdalan, Nicha Tulmethakaan, Donn J. Colby, Nitiya Chomchey, Peeriya Prueksakaew, Suteeraporn Pinyakorn, Rapee Trichavaroj, Julie L. Mitchell, Lydie Trautmann, Denise Hsu, Sandhya Vasan, Sopark Manasnayakorn, Mark de Souza, Sodsai Tovanabutra, Alexandra Schuetz, Merlin L. Robb, Nittaya Phanuphak, Jintanat Ananworanich, Timothy W. Schacker, Ashley T. Haase, on behalf of the RV254/SEARCH 010 Study Team

**Affiliations:** 1 Department of Microbiology and Immunology and; 2 Division of Infectious Diseases and International Medicine, Department of Medicine, University of Minnesota, Minneapolis, Minnesota, USA.; 3 Department of Infectious Disease and Vaccines, Merck & Co. Inc., Rahway, New Jersey, USA.; 4 Division of Biostatistics, School of Public Health, University of Minnesota, Minneapolis, Minnesota, USA.; 5 Institute of HIV Research and Innovation, Bangkok, Thailand.; 6 SEARCH Research Foundation, Bangkok, Thailand.; 7 Department of Medicine and; 8 Center of Excellence in Vaccine Research and Development (Chula Vaccine Research Center),; 9 Faculty of Medicine, Chulalongkorn University, Bangkok, Thailand.; 10 US Military HIV Research Program, Walter Reed Army Institute of Research, Silver Spring, Maryland, USA.; 11 Henry M. Jackson Foundation for the Advancement of Military Medicine Inc., Bethesda, Maryland, USA.; 12 Vaccine and Gene Therapy Institute, Oregon Health and Sciences University, Beaverton, Oregon, USA.; 13 Department of Surgery, Faculty of Medicine, Chulalongkorn University, Bangkok, Thailand.; 14 Armed Forces Research Institute of Medical Sciences, Bangkok, Thailand.; 15 Amsterdam University Medical Centers, Department of Global Health, Amsterdam Institute for Global Health & Development, Amsterdam, Netherlands.; 16 The RV254/SEARCH 010 Study Team is detailed in Supplemental Acknowledgments.

**Keywords:** AIDS/HIV, T cells

## Abstract

Productively infected cells are generally thought to arise from HIV infection of activated CD4^+^ T cells, and these infected activated cells are thought to be a recurring source of latently infected cells when a portion of the population transitions to a resting state. We discovered and report here that productively and latently infected cells can instead originate from direct infection of resting CD4^+^ T cell populations in lymphoid tissues in Fiebig I, the earliest stage of detectable HIV infection. We found that direct infection of resting CD4^+^ T cells was correlated with the availability of susceptible target cells in lymphoid tissues largely restricted to resting CD4^+^ T cells in which expression of pTEFb enabled productive infection, and we documented persistence of HIV-producing resting T cells during antiretroviral therapy (ART). Thus, we provide evidence of a mechanism by which direct infection of resting T cells in lymphoid tissues to generate productively and latently infected cells creates a mechanism by which the productively infected cells can replenish both populations and maintain two sources of virus from which HIV infection can rebound, even if ART is instituted at the earliest stage of detectable infection.

## Graphical abstract

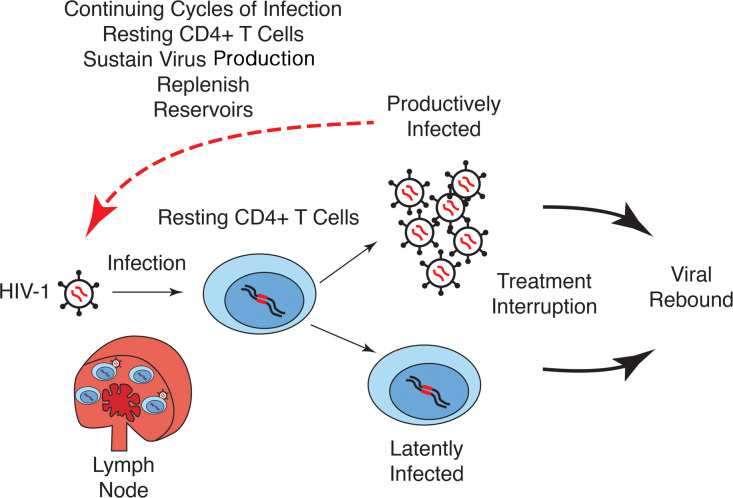

## Introduction

From the beginning of HIV research, HIV has been mainly propagated in vitro in tissue cultures of activated CD4^+^ T cells (1), leading to the prevailing view that activated CD4^+^ T cells are the major source of virus production in vivo, a conclusion supported by studies of the dynamics of the response to antiretroviral therapy (ART) in which the rapid decrease in virus parallels the decrease in activated infected cells in peripheral blood and lymphoid tissues (LT) (2, 3). Latently infected cells are thought to arise from infection of CD4^+^ T cells activated during an immune response, when the activated T cells, like their uninfected counterparts, return to an immunologically resting state (4), or from infection of activated CD4^+^ T cells in a narrow time window in the transition to resting memory cells (5), thereby establishing the reservoir in which HIV persists (6–10) as a barrier to cure (11–13).

How quickly are these populations of productively and latently infected cells established, and do both populations arise from infection of activated CD4^+^ T cells, as just described? We and others have investigated the in vivo origins of productively infected cell populations in the SIV nonhuman primate model and have shown in the earliest stage of infection following vaginal transmission that the SIV RNA^+^ CD4^+^ T cells initially detected at the portal of entry and in LT to which virus spreads were surprisingly not activated but rather had a resting phenotype (14). Furthermore, we showed that the infection of resting T cells was correlated with initial conditions of susceptible target cell availability predominantly limited to resting CD4^+^ T cells (15, 16) and that the infected resting T cells largely accounted for virus production in the earliest stages of SIV infection (17). While the phenotype of the cells in the latent reservoir was not determined, the latent reservoir in which SIV persists and from which infection rebounded when ART was discontinued is also established in the eclipse phase of SIV infection before virus is detectable in the circulation (18).

Could the initial events in HIV infection be like those in SIV infection, in which the preponderant target cell availability of resting CD4^+^ T cells dictates establishment of productive infections in HIV-infected resting T cells? And, if so, could infected resting T cells, rather than infected activated T cells, be the source of the latently infected cell population? The RV254/SEARCH 010 study (ClinicalTrials.gov NCT00796146) of acute HIV infection, including the earliest detectable stage of infection (Fiebig I), provided an opportunity to answer this question. In the RV254 study, participants with acute HIV were recruited at voluntary counseling and testing sites in Bangkok, Thailand. Blood samples of clients presenting for voluntary counseling and testing were screened by nucleic acid and/or sequential immunoassay within 1– 2 days of sample collection (19) to identify participants with acute HIV infection (Fiebig I–V). The vast majority of the participants with acute HIV infection consented to enrollment in a study of initiation of ART within a median time of 2 days after diagnosis, and a consenting subset of the cohort provided LT samples. These studies have documented (20) the rapid increase from rare cells in Fiebig I with integrated HIV DNA to large pools of infected cells by Fiebig II–III that are largely cleared after initiating ART. However, infected cells remained detectable in LT, and, despite the low frequency of HIV-infected cells when ART was initiated in Fiebig I, infection nonetheless rebounded when ART was interrupted (21). It thus seemed likely that analysis of LT samples obtained in Fiebig I prior to initiating ART might identify cell types in which productive and latent infections originate in the earliest detectable stage of HIV infection and sources of virus for recrudescent infection when ART was interrupted.

## Results

### Background on prior studies of HIV RNA^+^ cells in Fiebig I in RV254 LT samples.

Through the RV254 study, peripheral blood and tissue samples from individuals with acute HIV infection have been analyzed at more than 40 collaborating institutions worldwide thus far. The University of Minnesota research team focused first on detecting and quantifying HIV RNA^+^ cells in fixed lymph node (LN) biopsy samples, in which we showed that paradoxically there was greater persistence of HIV RNA^+^ cells in LT in the T cell zone when ART was initiated in Fiebig I compared with later Fiebig stages (22) and the persistence of HIV RNA^+^ cells during ART was correlated by at least one measure with variable antiretroviral drug penetration (23). In the studies reported here, we focused on Fiebig I to determine with the most sensitive methods available the origins of the first productively and latently infected cells in LT.

### Study design.

We designed our studies to test the hypotheses that the first productively and latently infected cells in Fiebig I would be resting CD4^+^ T cells, reflecting susceptible target cell availability and spatial proximity before activation of a detectable immune response at the earliest detectable stage of HIV infection and that HIV-producing resting CD4^+^ T cells would persist during ART. We phenotyped infected and uninfected cells from 8 participants in Fiebig I at the time of diagnosis. Their demographics, plasma viral load (pVL) at diagnosis and LN biopsy, CRF01 AE HIV infection, and criteria for Fiebig staging are described in Tables 1 and 2; the demographics and pVL in 4 participants after 2 or more years of ART are summarized in Table 3. We used DNAscope and RNAscope ISH with AE clade–specific probes to detect HIV DNA^+^ and RNA^+^ cells, respectively, and combined ISH with immunohistochemical (IHC) staining with antibodies to characterize cell type and activation status. We used antibodies against CD3 and CD4 for T cells and CD4^+^ T cells, respectively; CD68 for macrophages; Ki67 as a marker for late-stage activation and proliferation; and CD25 as a marker of early activation, based on the stereotypic program and kinetics following TCR-stimulated T cell activation (24) of rapid expression within a few hours of CD25 in CD4^+^ T cells that closely parallels CD69 expression and precedes expression of HLA-DR (25). To unequivocally identify and phenotype HIV-producing cells in LN sections, we used ISH and ELF97 to unambiguously identify virus-producing cells as cells with visible HIV virions at the resolution of immunofluorescence microscopy (26). These HIV-producing cells are hereafter referred to as HIV virus^+^.

### HIV RNA^+^ cells are CD3^+^Ki67^–^ T cells in LN in Fiebig I.

In the initial assessment of HIV RNA^+^ cells in Fiebig I LN, the HIV RNA^+^ cells were all CD3^+^ T cells in 7 of 8 participants; 98% of HIV RNA^+^ cells were CD3^+^ T cells in 1 participant, and 2% were CD68^+^ macrophages. All HIV RNA^+^ cells were Ki67^–^ (Table 4). Thus, HIV first infects and replicates almost entirely in T cells that were not activated and proliferating.

### HIV first infects and largely replicates in resting memory CD4^+^ T cells in Fiebig I LNs.

**Figure 1:**
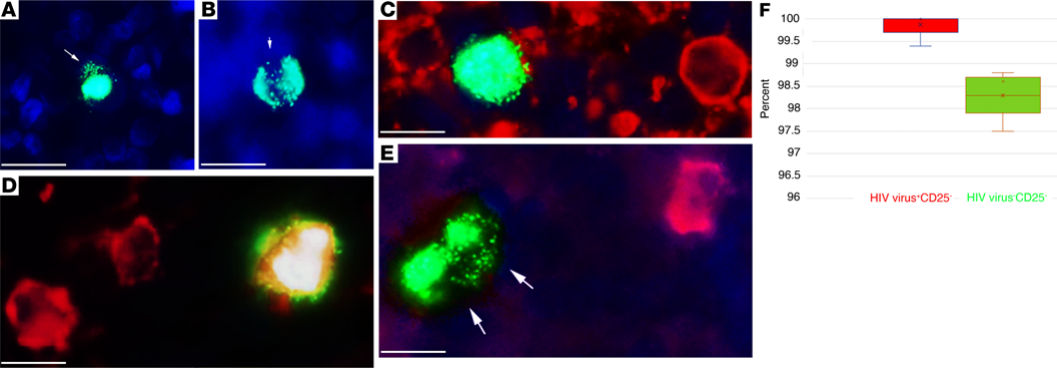
HIV-producing cells in Fiebig I are CD4^+^CD25^–^. ISH with ELF97 substrate identifies HIV-producing cells as cells with green virions visible against diffusely stained HIV intracellular RNA. Nuclei were counterstained blue with DAPI. (**A**) Example of a single productively infected cell. Arrow points to visible HIV virions. (**B**) Arrow points to visible virions shared by 2 cells, consistent with cell-to-cell spread. (**C** and **D**) HIV-producing cells are CD4^+^. Virions and intracellular RNA, green; CD4, red. (**C**) HIV-producing CD4^+^ cell with red/yellow CD4 staining visible at the cell’s margins. (**D**) Double-labeled red/yellow HIV-producing cell. (**E**) HIV-producing cells are CD25^–^. Virions and intracellular RNA, green; CD25, red; DAPI counterstain. Two HIV-producing (arrows) cell-CD25^–^ conjugates with visible HIV virions shared by the 2 cells (arrows) in a field with a CD25^+^ HIV^–^ cell. Scale bar: 10 mm. (**F**) Whisker plot of data in Table 5 from 5 participants, with percentages of HIV virus^+^ producing CD25^–^ cells (red; average 99.9%, range 99.4%–100%) and virus-uninfected CD25^–^ cells (green; average 98.3%, range 97.5%–100%). Scale bar: 10 µm.

The HIV DNA^+^ cells detected in Fiebig I LN in 8 of 8 participants were uniformly resting memory CD45RO^+^CD25^–^CD4^+^ T cells (Table 5). In 7 of 8 participants, in whom we were able to assess CD4^+^ T cell staining of cells visibly producing HIV virions (HIV virus^+^), 91% on average (range, 88%–100%) were CD4^+^ and 99% were CD25^–^ (Table 5). Because all HIV RNA^+^ cells were T cells, we interpreted the lower percentage of CD4^+^ HIV-producing T cells as likely reflecting decreased expression of CD4 in the productively infected cells. We illustrated single HIV-producing and cell-to-cell spread between HIV-producing cells in Figure 1, A and B; the CD4^+^ phenotype of HIV-producing cells in Figure 1, C and D; and cell-to-cell spread between HIV-producing CD25^–^ cells in Figure 1E. We thus attribute virus production in these LN samples in Fiebig I largely to HIV virus–producing CD4^+^ T cells with a CD25^–^ resting phenotype.

We further found that the predominance of productive infection in resting CD4^+^ T cells reflects target cell availability. In Fiebig I as the immune response is just developing, approximately 99% of the CD4^+^ T cells in these LN samples scored as CD25^–^ (Table 5). This circumscribed target cell availability correlated with the approximately 99% productive HIV infection in resting CD25^–^ T cells (Table 5 and Figure 1F).

### pTEFb expression and HIV production in resting T cells in Fiebig I LN.

Extensive literature exists on the many blocks to infection of resting CD4^+^ T cells in vitro that implies that there are critical differences between CD4^+^CD25^–^ T cells in vitro and resting T cells in LT in vivo that enable the resting cells to support HIV infection. These blocks (27) include coreceptor usage and levels, cortical actin restriction to entry (28), SAMHD1 (29), and pTEFb, the transcriptional elongation factor that plays an essential role in productive versus latent HIV infection (30–33). Given the limited availability of LT samples to identify critical differences in resting CD4^+^ T cells in vivo that enable the cells to support productive infection, we focused on pTEFb because of pTEFb’s essential role in HIV replication (33) with the prediction that resting CD4^+^ T cells in vivo in LNs must express pTEFb.

**Figure 2:**
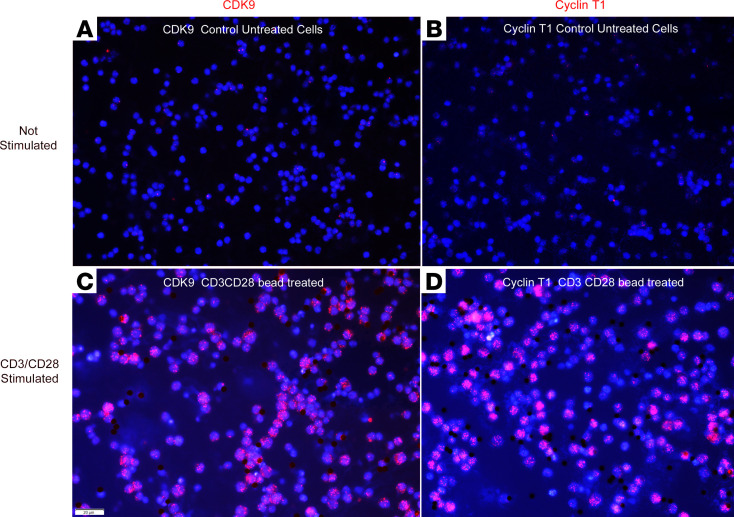
In vitro expression of pTEFb components CDK9 and cyclin T in PBMCs before and after stimulation. PBMCs from a person without HIV infection were cultured and (**A** and **B**) not stimulated or (**C** and **D**) stimulated with CD3/CD28 activator beads and stained with antibodies against CDK9 (left) or cyclin T1 (right). Nuclei were counterstained blue with DAPI. Without stimulation, CDK9 and cyclin T1 were not detectable in the nuclei. Cell stimulation induced expression of both CDK9 and cyclin T1 in a speckled pattern in the nuclei of stimulated cells. Scale bar: 20 μm.

**Figure 3:**
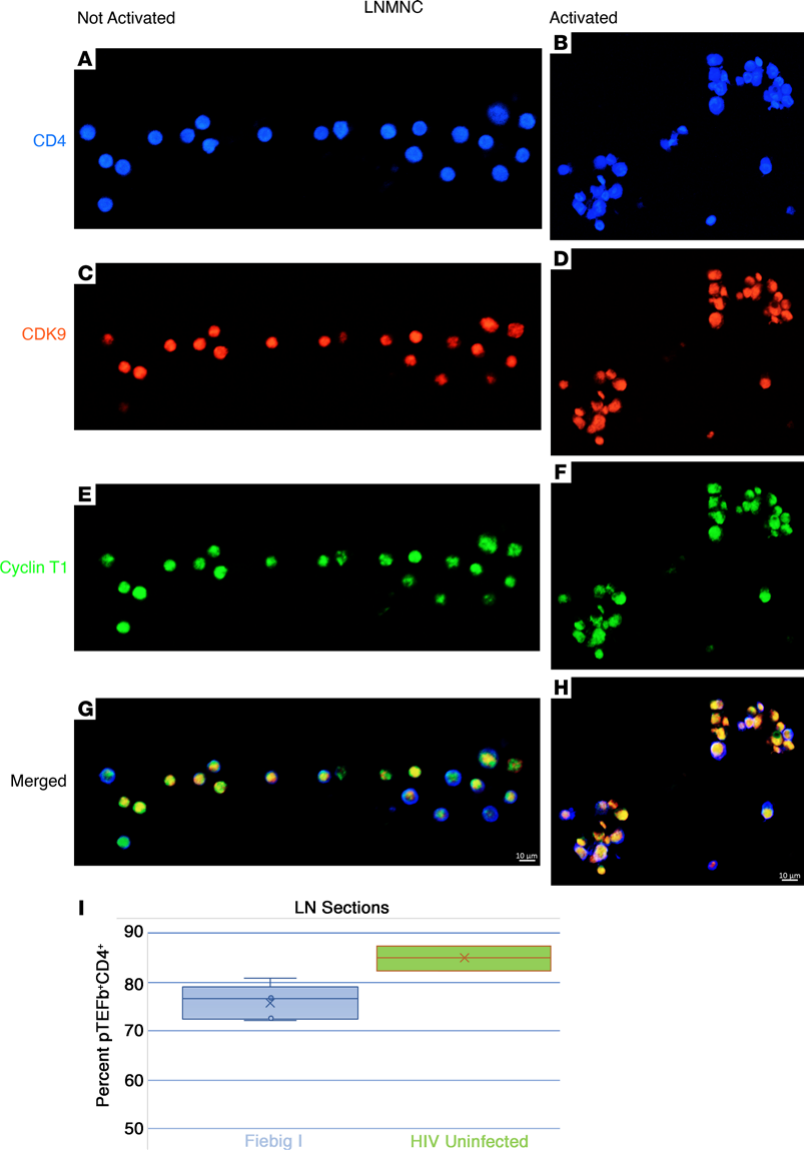
In vivo expression of pTEFb in CD4^+^ T cells in LNMNCs before and after activation with CD3/CD28 and in LN tissue sections. LNMNCs were either fixed immediately or grown for 2 days with CD3/CD28 beads and then fixed and pelleted. After Histogel added to the fixed cell pellet had solidified, the pellets were embedded in paraffin and sectioned and reacted with antibodies against CD4, CDK9, and cyclin T1. (**A** and **B**) CD4, blue; (**C** and **D**) CDK9, red; and (**E** and **F**) cyclin T1, green. (**G** and **H**) In the merged images, both before and after activation, pTEFb components CDK9 and cyclin T1 were detected as yellow- to orange-appearing nuclei in blue CD4^+^ T cells. Scale bar: 10 mm. (**I**) Whisker plot of data from Table 6 of pTEFb expression in CD4^+^ T cells in LN tissue sections from 5 participants in Fiebig I (average percentage, 76.7%; range, 72.1%–81%) and 2 individuals without HIV infection (average percentage, 84%; range, 82.5%–87.4%). Scale bar: 10 µm.

We first confirmed that CD4^+^CD25^–^ resting T cells in vitro did not express pTEFb until activated by showing that both pTEFb components CDK9 and cyclin T1 (30) were not expressed in unstimulated PBMCs from HIV-uninfected individuals but were detectable after stimulation with CD3/CD28 (Figure 2). By contrast, in vivo in LN mononuclear cells (LNMNCs), pTEFb components cyclin T1 and CDK9 were detected before activation in 64% of the cells and in 94% after activation (Figure 3, A–H). In LN tissue sections, 84% of CD4^+^ T cells in uninfected LN samples from 2 participants without HIV infection and approximately 77% of CD4^+^ T cells in LN sections from 5 Fiebig I participants were pTEFb^+^ (double positive for cyclin T1 and CDK9) (Table 6 and Figure 3I).

**Figure 4:**
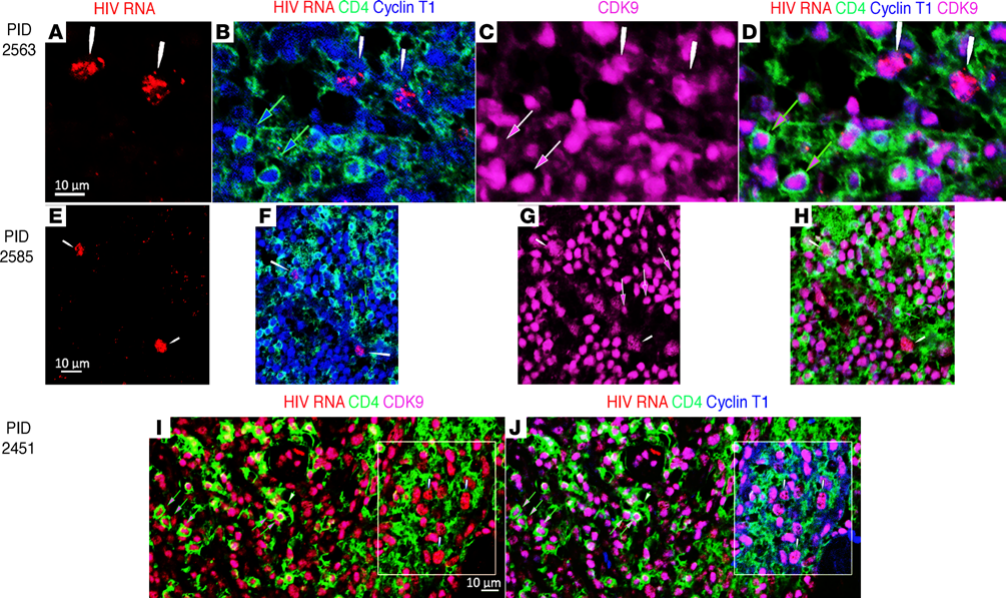
HIV infection of resting CD4^+^pTEFb^+^ populations in Fiebig I. (**A**–**H**) Participant ID (PID) 2563 and 2585. (**A** and **E**) White arrows track red HIV RNA^+^ cells in (**B** and **F**) green CD4^+^ cells with blue cyclin T1^+^ and (**C** and **G**) magenta CDK9^+^ nuclei. (**B** and **F**) Green-outlined blue arrows point to uninfected CD4^+^ T cells with cyclin T1^+^ nuclei. (**C** and **G**) Outlined magenta arrows point to CDK9^+^ nuclei in uninfected cells. (**D** and **H**) HIV RNA^+^ cells (white arrows) are green CD4^+^cyclin T1^+^ and CDK9^+^. Green outlined arrows point to uninfected cells with the same phenotype. (**I** and **J**) PID 2451. White arrows in the boxed region point to red HIV RNA^+^ cells in green CD4^+^ T cells with magenta CDK9^+^ and blue cyclin T1^+^ nuclei. Green outlined arrows point to uninfected CD4^+^CDK9^+^cyclin 1^+^ T cells. The outlined white arrow points to an uninfected CD4^+^ T cell whose nucleus is pTEFb^–^ (CDK9^–^cyclin T1^–^). Scale bar: 10 μm.

While the mechanism(s) is unknown for this basal state of pTEFb expression in vivo in CD4^+^ T cells in LNs, these results collectively show that there are large populations of susceptible target cells to support HIV replication in the LT microenvironment. We documented HIV infection of these pTEFb^+^ resting CD4^+^ T cells in Fiebig I LN by combining ISH detection of HIV RNA in CD4^+^ T cells with detection of cyclin T1 and CDK9 together in infected or uninfected cells. In 3 participants in Fiebig I, we tracked and colocalized all HIV RNA to CD4^+^ cells with pTEFb^+^ (cyclin T1^+^CDK9^+^) nuclei amid large numbers of uninfected CD4^+^ cells with pTEFb^+^ nuclei (Figure 4).

### Infection of resting T cells in Fiebig I and the origin of latently infected cells.

In the original model of latent infection of CD4^+^ T cells, latently infected cells were hypothesized to arise from infected activated cells as the cells returned to an immunologically resting state (4, 5). We show here that in Fiebig I prior to immune activation the only transcriptionally active and productive infections detectable are in resting CD4^+^ T cells, and, thus, this model cannot account for the origins of a latently infected cell population in the earliest detectable stage of HIV infection. Rather, because we show HIV replicates in Fiebig I in resting CD4^+^ T cells as essentially the only target population available, we hypothesized that the latently infected cell populations in Fiebig I LNs are also directly established as latent infections in resting CD4^+^ T cells.

To test this hypothesis, we assumed that latently infected cells might be quite rare in Fiebig I and therefore devoted the whole of approximately 10^7^ cells in the available LN cell suspensions to an improved ultrasensitive immunoassay with a dynamic range of 0.001–3 pg/mL and detection as little as 1 fg p24/mL (34) to maximize the probability of detecting both ongoing productive infections before stimulation and a small reactivatable population after stimulation. We isolated CD4^+^CD25^–^ cells from 3 Fiebig stage I participants, cultured the cells with and without stimulation with CD3/CD28 activator beads and with an integrase inhibitor, and prepared lysates for the ultrasensitive assay. We detected p24 production from ongoing replication in unstimulated cells at very low levels that after stimulation increased 5- to more than 30-fold, as evidence of a small reactivatable latently infected CD4^+^CD25^–^ population in 2 of the 3 Fiebig I samples (Table 7). In Fiebig I participant 2540, the high levels of p24 prior to stimulation from ongoing HIV productive infection did not increase after stimulation. This result is consistent with a relatively small latently infected population that even after stimulation would be undetectable against the high background of ongoing replication. Thus, while we were limited in availability of samples, as proof of principle, HIV demonstrably establishes a small reactivatable population of latently infected cells by infection of CD4^+^CD25^–^ resting T cells in Fiebig I.

### HIV-producing cells persist despite ART initiated in Fiebig I.

**Figure 5:**
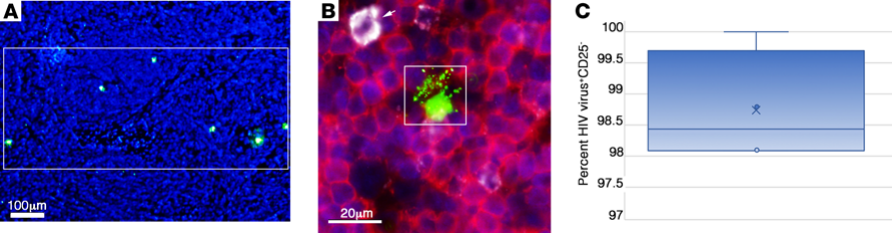
Persistence of HIV-producing resting CD4^+^ T cells during ART initiated in Fiebig I. (**A**) Low-magnification (original magnification, ×10) view of green HIV-producing cells in LN tissues after 24 months of ART initiated in Fiebig I. The rectangle encloses 6 HIV^+^ cells. Scale bar: 100 μm. (**B**) HIV RNA in the cell and cell fragments and in HIV virions is shown in green, and CD4^+^ cells are red with blue nuclei. The cell within the rectangle shows orange staining areas, indicating CD4^+^, and is surrounded by virions and clumps of cell-associated virions. Field of CD4^+^CD25^–^ cells except for 1 clearly CD4^+^CD25^+^ cell (white arrow). Scale bar: 20 μm. (**C**) Whisker plot of data from Table 8 showing that an average of 98.8% (range, 98.1%–100%) of the HIV-producing cells in 4 participants who initiated ART in Fiebig I were CD25^–^.

The potential implications of directly establishing HIV-producing and latently infected cells are first to create a mechanism for continuing replenishment from virus produced by resting T cells; and, second, potentially 2 sources of virus to reignite infection with treatment interruption. Participant 2540, described with a relatively larger population of productively infected compared with latently infected cells, is an example of the potential importance of persistent productive infections in viral rebound. We had shown a paradoxical persistence of HIV RNA^+^ cells despite initiation of ART in Fiebig I (22) and now asked whether the persistent HIV RNA^+^ cells might be HIV-producing resting CD4^+^ T cells as a second source of virus for recrudescent infection. We screened LN tissues from 4 participants after 96 to 240 weeks of ART initiated in Fiebig I with undetectable pVL <20 copies/mL (Table 3) and detected HIV-producing cells (Figure 5A) in all 4 participants, with an average frequency of 3.2 × 10^5^/g. The HIV-producing cells were CD4^+^, and the infected cells and surrounding corona of virions overlaid uninfected CD4^+^ T cells in regions of high CD4^+^ T cell density (Figure 5B). While these regions might have a few CD4^+^CD25^+^ T cells, these activated CD4^+^ T cells were not infected (Figure 5B) and 99% of the productively infected cells in the LNs of the 4 participants were CD4^+^CD25^–^ (Figure 5C and Table 8).

### Limitations of the study and impact of the pandemic.

The major limitation of our study was availability of LN samples, reflecting the collaborative overall design of the RV254 project, dispersal of samples, and the COVID-19 pandemic’s affect on continued patient accrual. Future availability of samples would enable analysis to strengthen support for the major findings as well as a contemporary transcriptomic analysis of the resting CD4^+^ T cells in LT and comparison with current measures of the HIV reservoir.

## Discussion

In Fiebig I, prior to immune activation in response to infection, HIV interacts with susceptible target cells in LT that are nearly exclusively resting CD4^+^ T cells. Like in SIV (14–16), we show that HIV can directly infect these resting CD4^+^ T cells to establish a self-propagating and expanding infection in Fiebig I as the prelude to systemic infection.

Because a self-propagating and expanding infection requires virus production, we used an unambiguous assay to document HIV-producing CD4^+^CD25^–^ T cells as the nearly exclusive source of virus in Fiebig I. We also focused on pTEFb as essential for virus production, with the hypothesis that pTEFb would be expressed in CD4^+^CD25^–^ T cells in vivo in LT in contrast to resting T cells in vitro (33), and found that HIV replicates in resting T cells in vivo that do express pTEFb in LT. Like the origins of latent infections by direct infection of resting CD4^+^ T cells, we speculate that the permissiveness of ostensibly resting T cells to direct infection and establishment of both productive and latent infections is related to the LT environment of cell-cell contact and interactions and secreted cytokines and factors (35–38), as has been described in cell culture models of direct establishment of latent infections in resting T cells.

The general view of the origins of latently infected resting memory CD4^+^ T cells is that they arise from infection of CD4^+^ T cells activated during an immune response as the activated T cells return to an immunologically resting state (4), but in Fiebig I, prior to immune activation in response to evolving infection, we showed that target cell availability in LN tissues was essentially limited to resting CD4^+^ T cell populations. Thus, the latently infected and reactivatable resting CD4^+^ T cell populations established in Fiebig I most plausibly arose from direct infection of this population.

We show herein how directly establishing HIV-producing and latently infected cells through infection of resting CD4^+^ T cells creates a mechanism to continually replenish both populations not only in Fiebig I, but also at any stage of infection. We also showed the potential for viral rebound when treatment is interrupted from HIV-producing resting CD4^+^ T cells that persist during ART as well as from reactivation of virus production from latently infected cells. We documented the persistence of HIV-producing resting CD4^+^CD25^–^ cells after about 2–4 years of ART initiated in Fiebig I. HIV-producing resting T cells persist during ART in substantial numbers. Taking 700 grams as the total weight of LT in a 70 kg person (39), the average of approximately 300,000 HIV-producing resting CD4^+^ T cells translates to a population of more than 200 million HIV-producing cells immediately available to reestablish systemic infection when treatment is interrupted, which could well have contributed to viral rebound, despite initiating ART in the earliest possible stage of infection (21).

While the evidence for and implications of direct infection of resting CD4^+^ T cells were derived from studies of Fiebig I LN, the major conclusion of the paper is based on the general principle that target cell availability and spatial proximity will dictate and predict the types of cells HIV infects. Thus, productive infection of resting T cells is expected to predominate whenever and wherever resting CD4^+^ T cells predominate in LT. While Fiebig I was close to a pure example to test this principle, it should hold independent of Fiebig stage, including later stages of infection, where, despite associated general immune activation, 99% of CD4^+^ T cells were Ki67^–^ in studies of CD4^+^ T cell populations in LT before and after initiating HAART (40). This principle also predicts that HIV-productive infections should track the site and availability of target cells that accompany an evolving and continuing immune response, which will increase the frequency of CD4^+^ T cells and T follicular helper (Tfh) cells in B cell follicles. Indeed, we have shown uninfected CD4^+^CD25^–^ Tfh cells do increase in frequency in hyperplastic B cell follicles, with a corresponding increase in the frequency of HIV-producing Tfh cells in accordance with the principle of availability and spatial proximity (our unpublished observations). However, uninfected resting CD4^+^CD25^–^ T cell targets and HIV-producing resting T cells still predominate, now located in or at the B cell follicle interface, and these HIV-producing resting CD4^+^ T cells are the predominant population that persists after treatment. Additional future studies will be needed to (a) determine the roles and relevance at all stages of HIV infection of resting CD4^+^ HIV-producing cells to the establishment, maintenance, and persistence of HIV reservoirs from which infection rebounds on treatment interruption and (b) identify ARVs that concentrate in LT (23) and combinations of ART and broadly neutralizing antibodies (41) and other treatment modalities that fully suppress continued propagation of HIV in resting CD4^+^ T cells to develop new strategies to effect a functional cure.

## Methods

### Processing of LN tissues.

LN from participants in the RV254 studies were cut in half. One half was disaggregated into a LNMNC cell suspension largely for studies at collaborating institutions, and one half was fixed in 4% paraformaldehyde/PBS for 24 hours. The fixed LN tissues samples were thereafter washed 3 times in 80% ethanol and embedded in paraffin, and the entire block was cut into 5-micron sections, mounted on Epic Plus Scientific microscope slides (Creative Waste Solutions), and heated at 60°C for 1 hour.

### RNAscope/DNAscope.

Every fifth section of the block was dewaxed in xylenes for 10 minutes (twice) and placed in 100% ethanol (twice) for 5 minutes before air drying. RNAscope 2.0 Red (ACD) was employed as previously described (42), using the HIV clade AE RNA probe (ACD, catalog 446551) or AE DNA probe (ACD, catalog 444011).

### RNAscope or DNAscope with immunohistochemistry/immunofluorescence.

RNAscope and DNAscope were employed using the 2.5 Brown Kit (ACD). After completing amp 6, slides were labeled with Opal 570 for 10 minutes. Slides were microwaved in their appropriate antigen retrieval buffer for 45 seconds at full power before dropping down to 20% power for 10 minutes. After cooling, samples were blocked in Sniper Blocking solution (Biocare Medical) for 30 minutes before adding the primary antibody, diluted in Divinci Green (Biocare Medical) overnight at 4 degrees. After washing slides in TBST, slides were incubated with opal polymer HRP mouse + rabbit (Ms +Rb, Akoya Biosciences) for 20 minutes. Opal 520 (Akoya Biosciences) was added for 20 minutes before adding DAPI and mounting. Reagents used are provided in Table 9.

### RNAScope detection of HIV-producing cells and phenotyping for CD4 and CD25.

HIV-producing cells were detected by incubating tissues at 40^°^C overnight with AE probe and then using the RNAscope 2.5HD detection reagent red kit (322360, ACD) and protocol, with the exception that after amplification 6 step, the tissue sections were washed twice for 2 minutes in wash buffer and then incubated with a 1:10 dilution of ELF97 phosphatase substrate (E6600, ELF immunohistochemistry kit, Invitrogen by Thermo Fisher Scientific) for 10 to 20 minutes. In the double-label phenotyping analyses, following the ELF97 step, tissues sections were twice washed in TBS for 5 minutes and blocked by background Sniper (BS966L, Biocare Medical) for 45 minutes, before incubating the tissue sections at 4°C overnight with anti-CD4 antibody (1:25 dilution, ab133616, Abcam) or CD25 (4C9) (1:20 dilution, 125M-16, Cell Marque). CD4^+^ T cells were subsequently detected by washing the tissue sections twice for 5 minutes in TBS; incubating for 2 hours at ambient temperature with Alexa Fluor donkey anti-rabbit 555 1:200 (A31572, Invitrogen) and DAPI for 5 minutes; and then cover slipping the tissue sections with Aqua-Polymount (18-606-20, Polysciences). CD25^+^ cells were detected after the TBS wash step by blocking with 3% H_2_O_2_ for 10 minutes, subsequent incubation with Opal Polymer HRP Ms+Rb for 10 minutes, washing twice for 5 minutes in TBS and Opal 570 reagent 1:100 (NEL810001KT, Akoya Biosciences) for 10 minutes, before washing, DAPI staining, and mounting as described for CD4. Slides were stored at 4°C until analyzed as follows. The entire section was scanned under WU illumination at ×40 to detect and capture images of HIV-producing cells, followed by images of CD4^+^ or CD25^+^ cells at the same location using the red filter. Images of the ELF97-HIV–producing cells and CD4 or CD25 were merged in Photoshop and single-positive HIV-producing and double-positive CD4^+^ or CD25^+^ were enumerated manually. In the triple-label phenotypic analysis, after detection of HIV-producing cells with RNAscope and ELF97, tissues sections were washed twice in TBS for 5 minutes, and CD25^+^ cells were detected first as described above. After the Opal 570 step, tissue sections were washed twice for 5 minutes in TBS and blocked with Opal Antibody Diluent/Block (Akoya, ARD1001EA) for 30 minutes, followed by detection of CD4^+^ cells by incubating tissue sections in the above blocking buffer at 4°C overnight. The tissue sections were then washed twice for 5 minutes in TBS, incubating for 2 hours at ambient temperature with Alexa Fluor donkey anti-rabbit 488 (1:200, Thermo Fisher Scientific, A-21206), stained with DAPI for 5 minutes, and then cover slipped with Aqua-Polymount. Slides were scanned under WU illumination at ×40 to first detect and capture images of HIV-producing cells, followed by images of CD4^+^ and CD25^+^ cells at the same location using the green and red filter separately. Images of the ELF97-HIV–producing cells, CD4 and CD25 were merged in Photoshop and single-positive HIV-producing and CD4^+^ and CD25^+^ cells were enumerated manually.

### Expression of pTEFb (cyclin T1/CDK9) in PBMCs and Fiebig I LNMNCs with and without activation.

PBMCs from an individual without HIV infection were thawed and grown in culture for 2 days, and Dynabeads (Human T-Activator CD3/CD28) were added 1:1 to 1 of the 2 flasks of cells for 2 days. Cells were harvested, spotted on glass slides, and fixed for 24 hours, and CDK9 and CycT1 were detected by Opal 570, following the method discussed below for LNMNCs.

### LNMNCs.

Cells from LN suspensions were either fixed immediately or grown for 2 days with Dynabeads (Human T-Activator CD3/CD28 for T Cell Expansion and Activation, Thermo Fisher Scientific). They were fixed for 24 hours in 4% paraformaldehyde, pelleted, and washed. Histogel (Fisher Scientific) was added to the fixed cell pellet, solidified, and processed for paraffin embedding. Six-micron paraffin sections were cut, dewaxed with xylene, and transferred through graded ethanols followed by water. An Opal 4-color kit (Akoya Biosciences NEL810001KT) was used to detect CD4, CDK9, and CycT1. Slides were retrieved by microwaving in DIVA (Biocare Medical) at 20% power for 5 minutes. Slides were blocked, incubated with rabbit anti-CD4 (Abcam), washed, treated with anti-rabbit peroxidase, washed, and reacted with the fluorescent peroxidase substrate Opal 520. AR6 buffer was used for retrieval of CDK9 and CycT1. After blocking and incubation with mouse anti-CycT1 (Santa Cruz), Opal 690 was used for detection. Finally, after retrieval, blocking, and incubation with rabbit anti-CDK9, CDK9 was detected with Opal 570. After staining slides with DAPI and mounting with Aqua Polymount (Polysciences), a Zeiss LSM800 confocal was used to capture 4-color images at ×20.

### Detection of HIV RNA^+^ pTEFb^+^ CD4^+^ T cells.

Immunofluorescence staining for HIV RNA, CycT1, CDK9, and CD4 proteins was performed simultaneously on 6 μm thick LN sections using a modification of the method described in Zhang et al. (14, 15). Slides were dewaxed, treated with 1 μg/mL proteinase K, and hybridized to HIV antisense RNA probe labeled with digoxigenin. After using TSA and Opal 620 to detect HIV RNA, multiplex Fluorescent IHC was performed using an Akoya Opal Multiplex IHC kit (NEL84000/KT, Akoya Biosciences). Sections were heated for a minimum time between steps to remove previous antibodies and unmask the next antigen. Anti-CD4 (Opal 520) was followed by anti-CycT1 (Opal 690) and then anti-CDK9 (Opal 405). Each antigen was unmasked using its optimal buffer. Three to 5 sections were tested for each tissue and patient, and images were taken using a Zeiss LSM800 Confocal/Super-resolution Microscope at ×20. Each CD4 cell in a field was numbered and scored for the presence of nuclear CycT1 and CDK9 using single-color channel overlays.

### Detection of reactivatable latently infected CD4^+^CD25^–^ T cells by ultrasensitive p24 immunoassay.

Isolation of CD4^+^CD25^–^ cells from frozen suspensions was performed using negative selection with a Miltenyi CD4 human T cell isolation kit to which biotinylated CD25 was added in excess. Cells were cultured at 10^6^ cells/mL in RPMI containing 100 nM raltegravir. Half of the cells were stimulated with Human T Cell activator CD3/CD28 beads (Thermo Scientific, catalog 11161D) (43, 44) at a bead/cell ratio of 1:1. After 3–5 days, both cell cultures were lysed in PBS containing 0.5% Triton X-100 and 3% BSA, and the stimulated and unstimulated lysates were tested for HIV p24 by combined immunoprecipitation and digital ELISA (34).

### Statistics.

No statistical analyses are reported.

### Study approval.

All samples analyzed in this study were obtained with the written consent of participants using protocols approved by Institutional Review Boards and Ethics Committees at the Walter Reed Army Institute of Research (approval 1494), Chulalongkorn University (approval 220/51), and the University of Minnesota (approval 1604M87147). The archived tissues used as historical chronic controls were obtained from participants in an Institutional Review Board–approved study at the University of Minnesota (approval 00009301; ClinicalTrials.gov NCT04311177).

### Data availability.

Values for all data points in graphs are reported in the Supporting Data Values file.

## Author contributions

ATH, TWS, PJS, and SWW designed the studies and developed and validated the methodology. SWW, PZ, and BJH contributed to experimental design and new assays/tools, and GW performed the reactivation studies. CR provided statistical analysis. SWW, JA, and LD designed and performed the image analyses. ATH and TWS acquired funding. EK, CS, NT, DJC, NC, PP, SP, JLM, LT, DH, SV, MLR, NP, and JA managed participant recruitment, enrollment, follow-up, and overall conduct of the RV254 cohort. SC, SB, RT, SM, MDS, ST, and AS managed specimen collection, processing, and laboratory assays involved with Fiebig staging. ATH wrote the original draft of the manuscript. ATH reviewed and edited the manuscript with contributions from all coauthors.

## Supplementary Material

Supplemental data

Supporting data values

## Figures and Tables

**Table 6:**
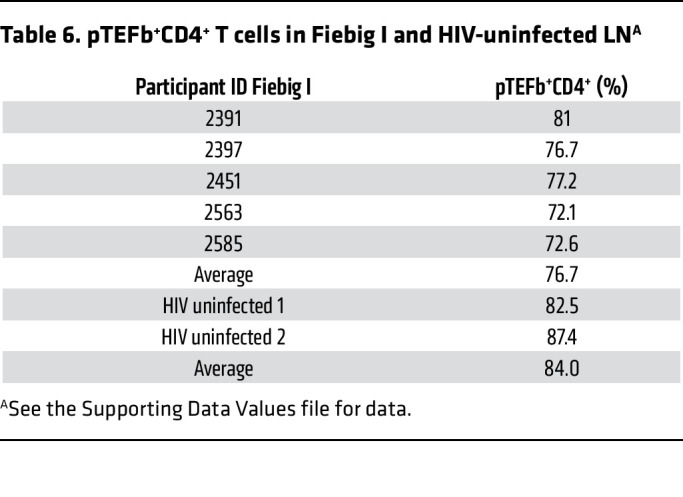
pTEFb^+^CD4^+^ T cells in Fiebig I and HIV-uninfected LN^A^

**Table 7:**
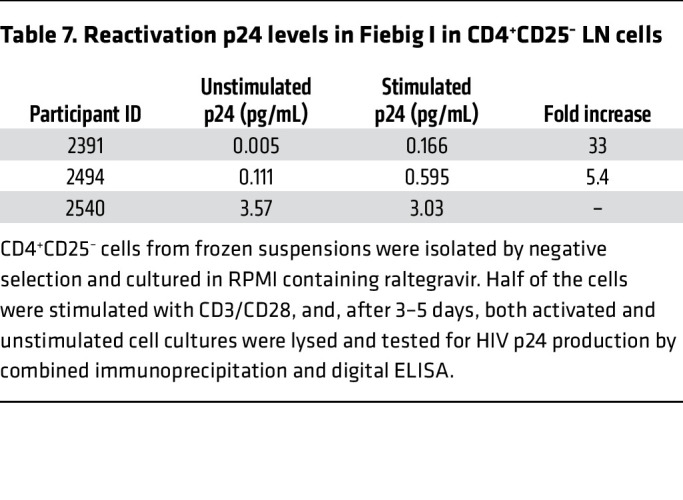
Reactivation p24 levels in Fiebig I in CD4^+^CD25^–^ LN cells

**Table 4:**
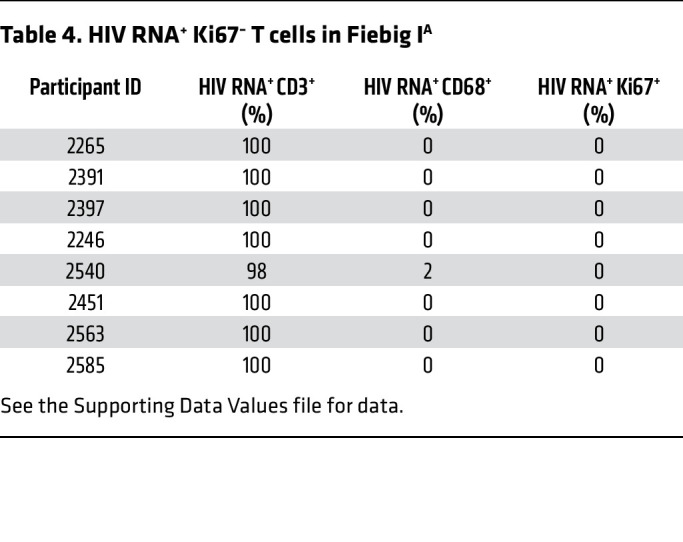
HIV RNA^+^ Ki67^–^ T cells in Fiebig I^A^

**Table 3:**
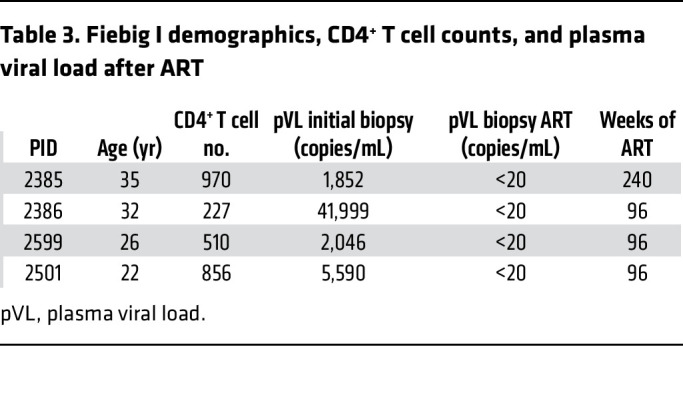
Fiebig I demographics, CD4^+^ T cell counts, and plasma viral load after ART

**Table 5:**
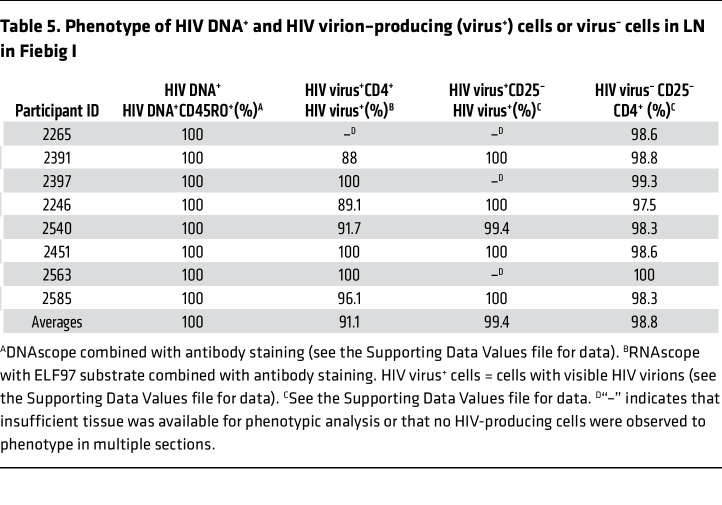
Phenotype of HIV DNA^+^ and HIV virion–producing (virus^+^) cells or virus^–^ cells in LN in Fiebig I

**Table 2:**
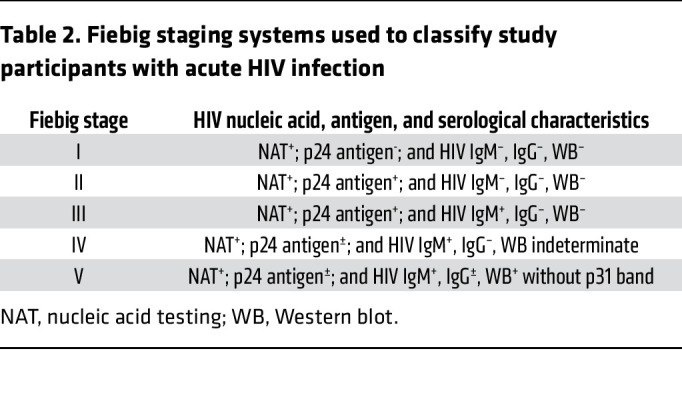
Fiebig staging systems used to classify study participants with acute HIV infection

**Table 1:**
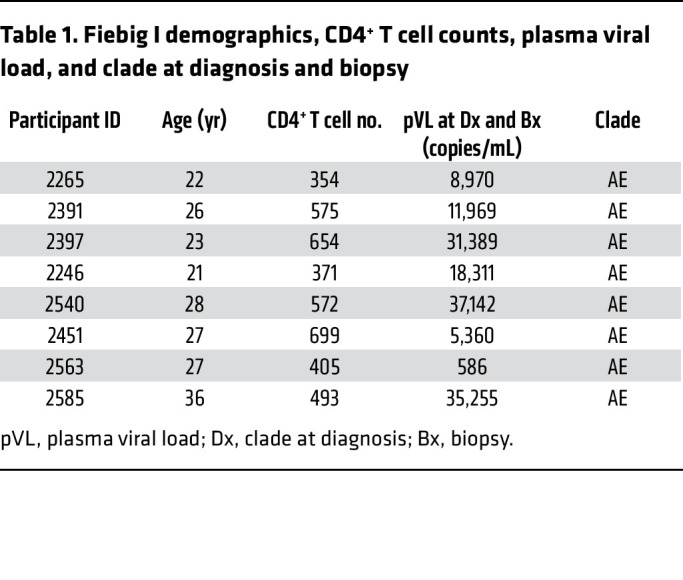
Fiebig I demographics, CD4^+^ T cell counts, plasma viral load, and clade at diagnosis and biopsy

**Table 9:**
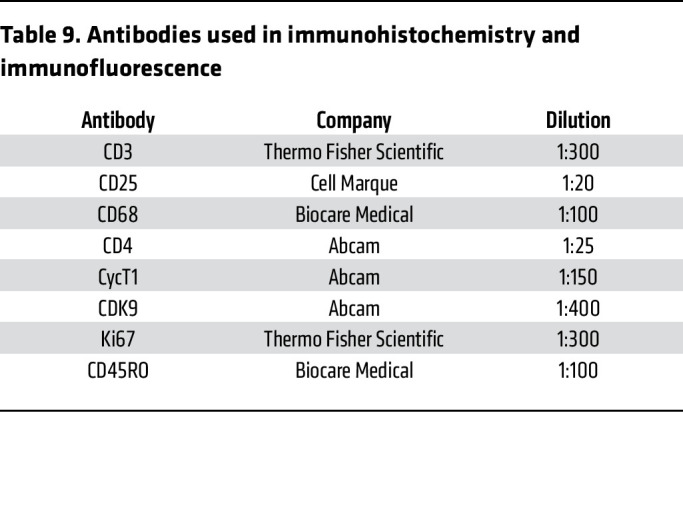
Antibodies used in immunohistochemistry and immunofluorescence

**Table 8:**
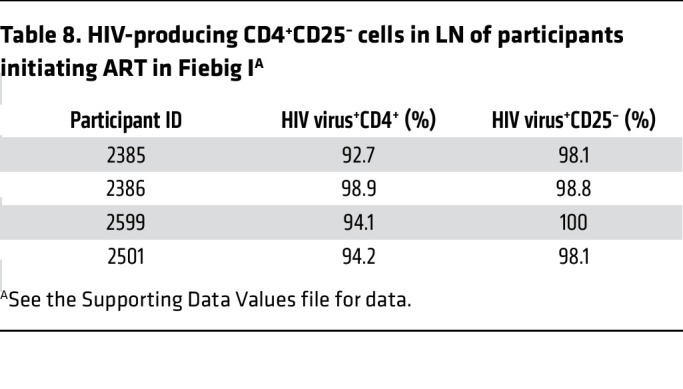
HIV-producing CD4^+^CD25^–^ cells in LN of participants initiating ART in Fiebig I^A^

## Version 1 09/21/2023

In-Press Preview

## Version 2 11/15/2023

Electronic publication
