# Supplementary material for: Initial productive and latent HIV infections originate in vivo by infection of resting T cells
Source: J Clin Invest. 2023 Nov 15;133(22):e171501. doi: 10.1172/JCI171501 (PMC10645380; doi:10.1172/JCI171501)
Supplement: Supplemental data [file jci-133-171501-s043.pdf]

RV254/SEARCH010 Consortium

SEARCH Research Foundation

Somchai Sriplienchan
Carlo Sacdalan
Pathariya Promsena
Eugene Kroon
Mark de Souza
Ratchapong Kanaprach
Nitiya Chomchey
Duanghathai Suttichom
Kultida Poltavee
Jintana Intasan
Tassanee Luekasemsuk
Hathairat Savadsuk
Peeriya Pruksakaew
Somporn Tipsuk
Suwanna Puttamsawin
Nisakorn Ratnaratorn
Chutharat Munkong
Nicha Tulmethakaan
Jarawee Wattana
Siriporn Sangthong
Rojchana Piyabanharn
Varaporn Unsombut
Varisara Prasertsin
Chonthicha Prasanphong
Nathornsorn Poltubtim

IHRI

Nittaya Phanuphak
Nipat Teeratakulpisarn

TRCARC

Opass Putcharoen
Yuwaree Pichitchok
Sasiwimol Ubolyam
Tippawan Pankam

Chulalongkorn University

Kiat Ruxrungtham
Netsiri Dumrongpisutikul
Ponlapat Rojnuckarin

Suthat Chottanapund
Supranee Buranapraditkun
Sukalya Lerdlum
Sopark Manasnayakorn
Rugsun Rerknimitr
Sunee Sirivichayakul
Phandee Wattanaboonyongcharoen

AFRIMS

Jessica Cowden
Alexandra Schuetz
Siriwat Akapirat
Nampueng Churikanont
Saowanit Getchalarat

MHRP

Sandhya Vasan
Lydie Trautmann
Trevor Crowell
Donn Colby
Suteeraporn Pinyakorn
Ellen Turk
Oratai Butterworth
Mark Milazzo
Leigh Anne Eller
Julie Ake
Merlin Robb
Leigh Anne Eller
Sarah Moreland

University of California San Francisco

Victor Valcour

Yale University School of Medicine

Serena Spudich

DAIDS Clinical Representative

CAPT Lawrence Fox
